# Beyond Abstinence: What Are We Really Measuring in Addiction Services?

**DOI:** 10.1192/bjo.2026.11535

**Published:** 2026-06-30

**Authors:** Mohammad Ali

**Affiliations:** Addictions Recovery Community (ARC-MK), Milton Keynes, United Kingdom

## Abstract

**Aims:**

Outcome measurement in addiction psychiatry commonly prioritises abstinence, attendance, and treatment completion. These endpoints may fail to capture meaningful clinical progress, particularly in outpatient settings where recovery is often non-linear. This evaluation aimed to compare routinely recorded service outcomes with patient-reported indicators of progress, and to assess their relationship to engagement and re-presentation.

**Methods:**

A retrospective service evaluation was conducted within an outpatient addiction service. Consecutive patients completing at least one follow-up appointment over a 6-month period were included (n=162). Routine outcome measures (attendance, substance use status, treatment completion) were extracted from clinical records. Patient-reported outcomes were obtained through brief structured questions recorded during routine reviews, focusing on stability, harm reduction, and help-seeking behaviour.

**Results:**

The cohort had a mean age of 42 years; 60% were male. Abstinence at last contact was documented in 28% of patients, while 71% demonstrated at least one patient-reported functional improvement. Improvements included reduced substance-related harm (46%), increased stability in housing or relationships (39%), and earlier help-seeking during relapse (33%). Attendance and abstinence showed weak correlation with patient-reported progress. Notably, 58% of patients who did not meet traditional “successful outcome” criteria reported clinically meaningful gains. Re-presentation rates within 6 months were lower among patients reporting functional improvements, regardless of abstinence status.

**Conclusion:**

Routinely used endpoints in addiction psychiatry may underestimate meaningful recovery and distort service effectiveness. Incorporating patient-centred and functional outcome measures alongside traditional metrics may better reflect real-world progress and inform service development in outpatient addiction care.

